# Saponin-based adjuvant-induced dendritic cell cross-presentation is dependent on PERK activation

**DOI:** 10.1007/s00018-022-04253-x

**Published:** 2022-04-09

**Authors:** Lisa G. M. Huis in ’t Veld, Nataschja I. Ho, Melisssa Wassink, Martijn H. den Brok, Gosse J. Adema

**Affiliations:** Radiotherapy and OncoImmunology Laboratory, Department of Radiation Oncology, Radboud Institute for Molecular Life Sciences, Radboud University Medical Center, Geert Grooteplein Zuid 32, 6525GA Nijmegen, The Netherlands

**Keywords:** Adjuvant, Dendritic cell, Cross-presentation, Saponin, ISCOM, Vaccine

## Abstract

Saponin-based adjuvants (SBAs) are promising new adjuvants that stand out as they not only enforce CD4 + T cell-mediated immunity and antibody responses, but also induce an unprecedented level of antigen cross-presentation by dendritic cells (DC) and subsequent CD8 + T cell activation. We discovered that SBA’s ability to boost cross-presentation depends on the induction of lipid bodies (LBs). Moreover, the MHCII^lo^CD11b^hi^ DC subset was identified to be most responsive to SBA-induced cross-presentation. The aim is to further unravel the mechanisms behind the induction of DC cross-presentation by SBAs. Here we show that SBAs specifically induce the PKR-like Endoplasmic Reticulum kinase (PERK) pathway and that SBA-induced DC cross-presentation is dependent on activation of the PERK pathway. PERK activation and LB formation are both crucial for SBA-induced cross-presentation and PERK inhibition has little or no effect on SBA-induced LB formation. SBA’s responsiveness, LB formation and PERK activation are specific for the MHCII^lo^CD11b^hi^ DCs. These findings contribute to understanding the pathways involved in SBA-induced cross-presentation and immune activation which will ultimately lead to the development of vaccines with improved efficiency and safety.

**Supplementary Information:**

The online version contains supplementary material available at 10.1007/s00018-022-04253-x.

## Introduction

Adjuvants are pivotal elements in the vaccines not containing live attenuated virus to boost immune responses and increase the vaccine’s efficacy. Most of the classical vaccine adjuvants induce a strong Th2 response leading to neutralizing antibody production. These adjuvants, however, are relatively poor inducers of strong cell-mediated immune responses. Especially for diseases such as cancer and viral infections, strong cellular immunity mediated by CD8 + killer T cells is necessary for vaccine efficacy. Dendritic cells (DCs) are crucial for CD8 + T cell activation via so-called cross-priming. Cross-priming of CD8 + T cells is dependent on the level of DC maturation (co-stimulatory molecules and cytokines) and DC antigen cross-presentation efficiency, the preferential shuttling of exogenous antigens to the MHC-I pathway resulting in specific MHC-I/peptide complexes on the cell surface. The importance of adjuvants enhancing cross-presentation by DCs has been described by Ho et al. [1].

Saponin-based adjuvants (SBAs) are promising new adjuvants that stand out as they not only enforce CD4 + T cell-mediated immunity and antibody responses, but also induce an unprecedented level of antigen cross-presentation by DCs and subsequent CD8 + T cell activation. Saponins are glycosides that can be found in many plants. A specific part of the saponins isolated from the South American soapbark tree are shown to have adjuvant activity [2]. To improve stability and safety, saponins are formulated into forty nanometer cage-like particles called immune stimulatory complexes (ISCOMs) which is a mix of saponins, cholesterol and phospholipids [3]. In our previous work, we have shown the strong adjuvant capacity of the saponin Fraction C (FC) and its corresponding ISCOM Matrix C in cancer [4]. The potency of SBAs is further highlighted by the efficacy and safety of the SBA-containing SARS-CoV-2 vaccine (NVX-CoV2373) in human phase III trials and its approval by the regulatory authorities [5–7]. The effectivity of other SBA-containing vaccines has further been shown for Malaria and Herpes Zoster infection [8, 9].

The molecular mechanisms of DC cross-presentation are still not fully understood. CD8α + DCs are considered to be the most potent cross-presenting DC subset without exogenous adjuvants, although multiple subsets have the capacity to cross-present under specific circumstances [10–12]. Two main pathways of antigen cross-presentation in DCs have been proposed: the cytosolic pathway and the vacuolar pathway [13]. In the cytosolic cross-presentation pathway, exogenous antigens are slowly degraded in endosomal compartments by enzymatic digestion at acidic pH. Antigens then gain access to the cytosol, where they are further degraded by the proteasome into peptides and then enter the classical MHC-I presentation route. In contrast, cross-presentation through the vacuolar pathway is proteasome independent, but sensitive to blockade of lysosomal proteolysis. Antigen processing and loading on MHC-I, therefore, only occurs in endocytic compartments. Previously, ER-associated degradation (ERAD) has also been shown to facilitate DC cross-presentation by enabling antigen dislocation [14–19]. ERAD is induced by the Unfolded Protein Response (UPR) and its activation leads to proteasomal degradation of unfolded proteins. The main function of the UPR is to restore protein homeostasis in case of accumulation of misfolded and unfolded proteins in the ER lumen [20]. The UPR consists of three signaling pathways, initiated by the ER stress sensors: inositol-requiring enzyme 1α (IRE1α), PKR-like Endoplasmic Reticulum kinase (PERK) and activating transcription factor 6α (ATF6α). The UPR gets activated upon ER stress, which can be caused by numerous factors from within and outside the cell, disturbing the ER protein-folding machinery.

We showed in previous work that the high level of cross-presentation induced by SBAs is critically dependent on the proteasome and that SBA-triggered antigen translocation and endosomal escape is preceded by endosomal acidification [21]. This suggests that SBA-induced cross-presentation has features of the cytosolic pathway of cross-presentation. Moreover, we have shown that the SBA-induced DC cross-presentation is independent of TLR4, MyD88, Trif, NLRP3 or IFNAR signaling, demonstrating that SBAs act independent of TLR activation [21].

Furthermore, our data demonstrated that monocytic MHCII^lo^CD11b^hi^ DCs, present in the with GM–CSF-cultured bone-marrow-derived DCs (BMDCs) in vitro and in the lymph nodes in vivo, are the immune cells that are most responsive to SBA-induced cross-presentation [21]. Moreover, we uncovered that SBA’s ability to boost cross-presentation depends on the induction of lipid bodies (LBs), which are cellular organelles that consist of neutral lipids, i.e., di- and triacylglycerols and sterol esters, surrounded by a phospholipid monolayer. LBs store lipids in conditions of nutrient surplus and also prevent lipotoxicity [22]. LBs are increasingly recognized to play a role in lipid metabolism, but also in immune regulation [21, 23–32]. Cross-presentation and lipid body (LB) induction both occurred specifically in the monocytic MHCII^lo^CD11b^hi^ DCs. Genetic and pharmacological interference with LB induction abrogated the SBA-induced cross-presentation both in vitro and in vivo, highlighting its importance in SBA activity [21]. The exact mechanism of LB induction leading to DC cross-presentation remains to be elucidated.

We now performed RNA expression profiling of SBA-treated and untreated DC subsets and identified the ER stress and the Unfolded Protein Response as dominant pathways induced by SBAs. The data showed that SBAs specifically induce the PERK pathway of the Unfolded Protein Response uniquely in the SBA-responsive MHCII^lo^CD11b^hi^ DC subset. We demonstrated that PERK activation is crucial for SBA-induced DC cross-presentation and subsequent CD8 + T cell activation. Furthermore, PERK inhibition did not prevent the induction of LBs by SBAs, thus PERK activation and LB formation are both crucial for SBA-induced cross-presentation. Understanding the pathways involved in SBA-induced cross-presentation and immune activation will ultimately benefit the development of vaccines with improved efficiency and safety.

## Materials and methods

### Mice

Female wild-type C57Bl/6 J mice were purchased from Charles River (Sulzfeld, Germany) and bone-marrow for DC cultures was used from 6- to 16-weeks-old animals. C57Bl/6 OT-IxCD90.1 + (Thy-1.1) mice were bred and held in house, and the spleen of female 8–12 weeks old animals was used for OT-I experiments. All mice were held under specified pathogen-free conditions in the Central Animal Laboratory (Nijmegen, the Netherlands). All animal experiments were approved by the Animal Experimental Committee of the Radboud UMC, and were performed in accordance with institutional, national and European guidelines.

### Primary cell culture of GM–CSF and Flt3-L DCs

**Fig. 1:**
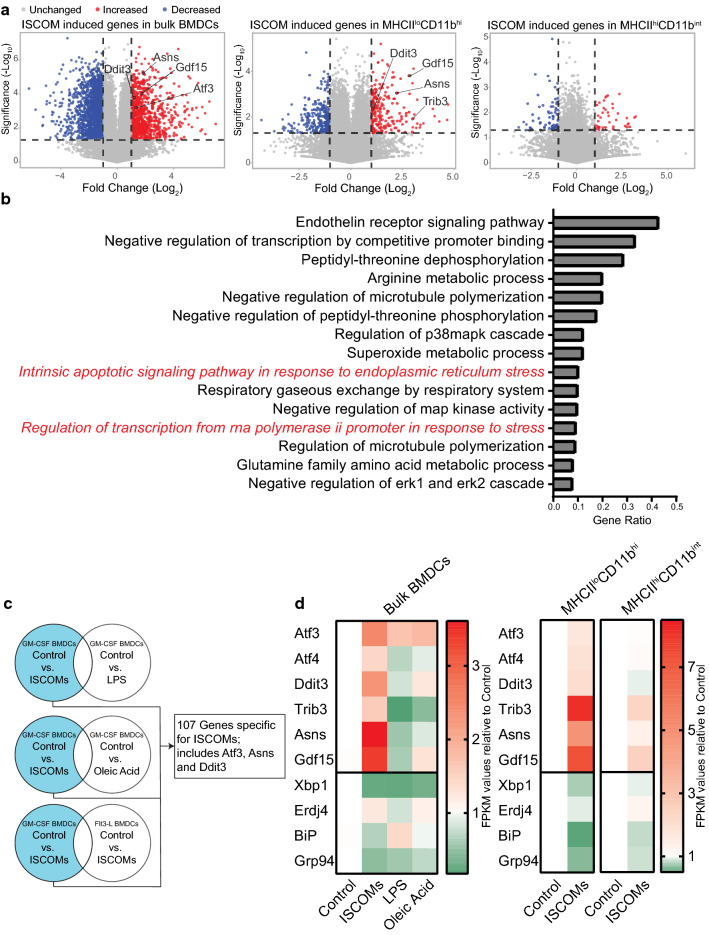
RNA sequencing shows that ISCOMs specifically induce the PERK pathway. RNA sequencing was performed with bulk GM*–*CSF-cultured BMDCs unstimulated or stimulated with ISCOMs, LPS or oleic acid. RNA sequencing was performed with Flt3-L-cultured BMDCs unstimulated or stimulated with ISCOMs. RNA sequencing was performed with sorted MHCII^lo^CD11b^hi^ and MHCII^hi^CD11b^int^ GM*–*CSF-cultured BMDCs unstimulated or ISCOM-stimulated. All data are based on FPKM values. Volcano plots of DEGs upon ISCOM stimulation vs. control with significantly upregulated genes in red and significantly downregulated genes in blue of bulk BMDCs (left), sorted MHCII^lo^CD11b^hi^ BMDCs (middle) and MHCII^hi^CD11b^int^ BMDCs (right). Significance shown in –log_10_ transformation with threshold *p* ≤ 0.05. Fold change shown in a log_2_ transformation with threshold ≤  − 2 or ≥ 2 (**a**). Gene ontology enrichment for Biological Processes of the significant DEGs by ISCOM treatment in MHCII^lo^CD11b^hi^ BMDCs using the String database (Thresholds *p* ≤ 0.05; fold change ≤  − 2 or ≥ 2; FDR ≤ 0.05). Top 15 processes are shown based on highest gene ratio (**b**). Combinatorial approach for DEGs by ISCOMs, but not by LPS or oleic acid in bulk GM*–*CSF-cultured BMDCs and not by ISCOMs in Flt3-L-cultured BMDCs (**c**). Unclustered heat maps of gene regulation of UPR genes in bulk (left) and sorted BMDCs (right); gene expression in FPKM values, relative to the control (**d**). RNA sequencing for bulk BMDCs was performed on 3 biological replicates and for sorted BMDC subset on 2 biological replicates

Bone marrow cells were flushed from the femur and tibia, and filtered using a 100 μm cell strainer (10282631, Corning Falcon). Erythrocyte lysis was performed by resuspending the cell pellet in cold ACK buffer (8.3 g/L NH_4_Cl, 1 g/L KHCO_3_, 37.3 mg/L EDTA in MQ, pH 7.2–7.4) for 1 min, after which cells were plated in 10 cm Petridishes (633180, Greiner) in complete RPMI medium (RPMI 1640 (42401042, Gibco), supplemented with 10% heat-inactivated fetal bovine serum (FBS; F7524–500ML, Gibco), 1% ultraglutamine (BE17–605E/U1, Lonza), 0.1% 2-mercaptoethanol (21985023, Gibco) and 1% penstrep (15140163, Gibco)). For GM–CSF-cultured BMDCs, 3–4 × 10^6^ cells were plated per dish and supplemented with 20 ng/ml recombinant murine GM–CSF (315-03, Peprotech) at the start of the culture and incubated at 37 °C with 5% CO_2_. Additional medium and GM–CSF were supplemented after 3 days and 6 days of culture to get at least 8.75 ng/ml GM–CSF. After 7 days of culture, the non-adherent cells were harvested and used for experiments (Protocol adapted from Lutz et al. [33]). For Clec9A+CD103+ Flt3-L-cultured BMDCs (only in Fig. 1c), 15 × 10^6^ cells were plated per dish and supplemented with 5 ng/ml GM–CSF and 200 ng/ml recombinant human Flt3-Ligand (Flt3-L; AF-300-19, Peprotech) at the start of the culture and incubated at 37 °C with 10% CO_2_. Additional medium, GM–CSF and Flt3-L were supplemented after 6 days of culture to get at least 5 ng/ml GM–CSF and 200 ng/ml Flt3-L. After 9 days of culture, non-adhering cells were harvested and re-plated with 3 × 10^6^ per dish with new medium and 5 ng/ml GM–CSF and 200 ng/ml Flt3-L. After 14 days of culture, non-adhering cells were harvested and used for experiments (protocol adapted from Mayer et al. [34]).

### DC sorting

Sorting of GM–CSF-cultured BMDC CD11c + MHCII^lo^CD11b^hi^ and CD11c + MHCII^hi^CD11b^int^ subsets was performed by staining for CD11c–APC (1:200, clone N418, 117310, Biolegend), MHCII–BV510 (I-A/I-E, 1:250, clone M5/114.15.2, 553142, Biolegend) and CD11b–A700 (1:200, Clone M1/70, 107636, Biolegend) diluted in FACS buffer (0.5% BSA 0.05% sodium azide in PBS) and incubated for 20 min at 4 °C. Staining was preceded by blocking Fc receptors for 10 min at 4 °C using anti-CD16/CD32 antibodies (1:800, clone 2.4G2, 553142, BD) diluted in FACS buffer. Cells were washed twice with FACS buffer, transferred through a 70 μm filter (340605, BD) and sorted on the FACS Aria system (BD biosciences) (protocol adapted from Helft et al. [35]).

### RNA sequencing and gene expression analysis

For the bulk DCs, GM–CSF DCs or Flt3-L DCs were harvested, MACS sorted for CD11c + cells with CD11c microbeads (130-052-001, Miltenyi Biotec) according to manufacturer’s instructions. CD11c + cells from GM–CSF DCs were untreated (control) or treated with Matrix C ISCOMs (400 ng/ml, MSD Animal Health, Boxmeer, the Netherlands) for 5 h, LPS (1 μg/ml, L4391, Sigma-Aldrich) for 6 h, or oleic acid (50 μM, O1008, Sigma-Aldrich) for 5 h. CD11c + cells from Flt3-L DCs were untreated (control) or treated with Matrix C ISCOMs (400 ng/ml) for 5 h. For bulk DCs, per condition 3 biological replicates were sequenced. For sorted DCs, GM–CSF DCs were harvested, sorted into CD11c + MHCII^lo^CD11b^hi^ and CD11c + MHCII^hi^CD11b^int^ subsets (see DC Sorting) and were untreated (control) or treated with Matrix C ISCOMs (400 ng/ml) for 5 h. For sorted DCs, per condition 2 biological replicates were sequenced. RNA was isolated using TRIzol™ Reagent (15596-018, Invitrogen), according to manufacturer’s instructions. RNA sequencing was performed by BGI Genomics (Hong Kong). The number of aligned reads were counted using feature count in the subread package (v.1.5.3) using the reference genome Gencode GRCm38 (v.M15) and data was normalized (TMM: trimmed mean of *M* values). All analyses shown are based on FPKM values.

Volcano plots show differentially expressed genes (DEGs) between control and ISCOM-stimulated BMDCs (bulk and CD11c + MHCII^lo^CD11b^hi^ and CD11c + MHCII^hi^CD11b^int^ BMDCs), were generated using VolcaNoseR [36]. DEGs were differentially expressed with significance *p* ≤ 0.05 and fold change compared to control ≤ − 2 or ≥ 2, with mean FPKM ≥ 1 in at least one of the compared conditions.

Gene Ontology was used to identify enriched Biological Processes of the significant DEGs by ISCOM treatment in CD11c + MHCII^lo^CD11b^hi^ DCs using the String database (string-db.org; Version 11.5) with thresholds *p* ≤ 0.05; fold change ≤ − 2 or ≥ 2; False Discovery Rate (FDR) ≤ 0.05 and with mean FPKM ≥ 1 in at least one of the compared conditions. Processes were rated and shown based on the Gene ratio of that pathway, calculated as Observed gene count/Total gene count.

Combinatorial approach for DEGs (threshold *p* ≤ 0.05 and mean FPKM ≥ 1 in at least one of the compared conditions) compared genes specifically regulated by ISCOMs (5 h), but not by LPS (6 h) or oleic acid (5 h) and not by ISCOMs in Flt3-L-cultured BMDCs, all in bulk BMDCs.

Unclustered heat maps were created with GraphPad Prism Version 8.0.1 to compare gene expression of UPR genes (gene choice based on literature research) in bulk and sorted BMDCs; gene expression in FPKM values, relative to the control.

### B3Z cross-presentation assays

Cross-presentation assays were performed using B3Z T cells. B3Z cells are CD8 + T cell hybridoma cells with a T cell receptor specific for SIINFEKL–MHC-I (H-2 Kb) complexes and an NFAT-LacZ reporter construct leading to β-galactosidase production upon T cell activation in a co-stimulation independent manner [37]. B3Z cells were cultured in IMDM medium (21980065, Gibco), supplemented with 5% heat-inactivated fetal bovine serum (F7524-500ML, Gibco), 1% ultraglutamine (BE17-605E/U1, Lonza), 500 μg/ml hygromycin B (10687010, invitrogen), 0.1% 2-mercaptoethanol (21985023, Gibco) and 1% penstrep (15140163, Gibco) at 37 °C with 5% CO_2_. B3Z assays were performed in complete RPMI medium. 8 × 10^4^ unsorted or sorted GM–CSF DCs were plated per well (U-Shaped-Bottom 96-well plate; 10360691, Corning costar) and incubated with chicken egg ovalbumin protein (80 μg/ml, OVA protein; LET0028, Lionex) Matrix C ISCOMs (400 ng/ml, MSD) or Fraction C saponin (800 ng/ml, MSD) in the absence or presence of the PERK inhibitor GSK2606414 (stock in DMSO, 5107, Tocris), the IRE1α inhibitor 4μ8C (stock in DMSO, 4479, Tocris), or the ATF6 inhibitor PF-429242 (S1P inhibitor; stock in DMSO; SML0667-5MG, Sigma-Aldrich) for 5 h (inhibitor concentration indicated in the figures). For the OVA peptide-pulsed cells, 5 ng/ml OVA peptide (SIINFEKL: OVA257-264; AS-60193-5, Anaspec) was added during the last 30 min. The medium was washed away, and 8 × 10^4^ B3Z cells per well were added and incubated for 18 h more. The cross-presentation of OVA protein or passive loading of the OVA peptide leads to SIINFEKL presentation in the MHC-I (H-2 Kb) molecule and subsequent β-galactosidase (LacZ) production by the activated B3Z cells, which is detected using 0.15 mM chlorophenolred-h-d-galactopyranoside (220588-250 mg, Sigma-Aldrich), 9 mM MgCl_2_, 0.125% NP40 and 7.5 mM DTT in PBS, leading to conversion into Chlorophenol red and Galactose causing a color change, and after 2–6 h incubation at 37 °C the absorbance was measured using a photospectrometer at 595 nm.

### Cell counting kit-8 (CCK8) assay

To measure cell metabolic activity as a measure for cell viability, 8 × 10^4^ GM–CSF BMDCs per well (U-shaped-bottom 96-well plate; 10360691, corning costar) were incubated with 400 ng/ml Matrix C ISCOMs in the absence or presence of the PERK inhibitor GSK2606414 for 5 h at 37 °C with 5% CO_2_. The cells were washed, the medium was replaced and incubated for 18 h more. To read out the assay, 100 μl new medium and 10 μl CCK8 reagent (96992-3000TESTS-F, Sigma-Aldrich) were added to each well and after 1–3 h incubation at 37 °C the absorbance was measured using a photospectrometer at 450 nm. The relative metabolic activity was calculated as (treatment—blank)/(control—blank) × 100%.

### RNA isolation and RT-qPCR

For RNA isolation, 2–5 × 10^5^ unsorted and sorted GM–CSF DCs were incubated with Matrix C ISCOMs (400 ng/ml), the PERK inhibitor GSK2606414 (10 μM) or Thapsigargin (50 nM, stock in DMSO, T9033, Sigma-Aldrich), for 5 h at 37 °C with 5% CO_2_. Control samples are treated with the same amount of DMSO (D8418, Sigma) as the PERK inhibitor or Thapsigargin. Total RNA was isolated either using the Quick-RNA Miniprep Kit (ZY-R1055, Zymo Research) with DNAse treatment on the column or using TRIzol™ Reagent (15596-018, Invitrogen) in combination with DNAse I (18068015, Invitrogen) treatment, all according to manufacturer’s instructions. RNA was quantified using the Nanodrop spectrophotometer. RNA was reverse-transcribed into cDNA by first incubating 500 ng of RNA with random p(dN)6 primers (11034731001, Roche) and dNTPs (NU-0020-50, Eurogentec) for 10 min at 65 °C, cooling down on ice and subsequent incubation with First-Strand Buffer, DTT, M-MLV Reverse Transcriptase (28025-021, Invitrogen) and RNAsin Ribonuclease Inhibitor (N2515, Promega) for 10 min at 25 °C, 50 min at 37 °C, 15 min at 70 °C and cooling down on ice. Reactions contained diluted cDNA, murine forward (300 nM) and reverse primers (300 nM) and FastStart SYBR Green Master (4673484001, Roche) and RT-qPCR was performed according to manufacturer’s instructions using the CFX96 Real-Time PCR Detection System (Bio-Rad). Gene expression is shown as ddCT using Pbgd as reference gene. An overview of the murine RT-qPCR primers is shown in Table 1.

**Table 1:** Overview of murine RT-qPCR primers

Gene	Forward primer	Reverse primer
Pbgd (reference gene)	CCTACCATACTACCTCCTGGCTTTAC	TTTGGGTGAAAGACAACAGCAT
Atf3	CCATCCAGAATAAACACCTC	GCACTCTGTCTTCTCCTTTT
Atf4	CCAAGCACTTGAAACCTC	CTTTCAGATCCATTTTCTCC
Ddit3	CTGGAAGCCTGGTATGAGGAT	CAGGGTCAAGAGTAGTGAAGGT
Trib3	ATATCCTTTTGGAACGAGAG	AAGATGTAAAGGAGCCGAG
Asns	CACAAGGCGCTACAGCAAC	CCAGCATACAGATGGTTTTCTCG
Gdf15	CTCAGAACCAAGTCCTGACC	GACCCCAATCTCACCTCT
Xbp1 spliced	CTGAGTCCGCAGCAGGTGCAG	ACAGGGTCCAACTTGTCCAGAA
Xbp1 unspliced	CAGCACTCAGACTATGTGCA	ACAGGGTCCAACTTGTCCAGAA
Xbp1 total	TGGCCGGGTCTGCTGAGTCCG	ACAGGGTCCAACTTGTCCAGAA
Erdj4	TCAGAGCGACAAATCAAAAAGGC	CTATTGGCATCCGAGAGTGTTT
BiP	CAGGCTGGTGTCCTCTCTGG	CTCCCACAGTTTCAATACCAAGTG
Grp94	GTTCGTCAGAGCTGATGATGAA	GCGTTTAACCCATCCAACTGAAT

### OT-I cross-priming assays

OT-I mice have CD8 + CD90.1 + T cells expressing a transgenic T cell receptor specific for OVA peptide (SIINFEKL: OVA257-264) presented in the MHC-I (H-2 Kb) molecule. The spleen from OT-I mice was disrupted in 2%FBS in PBS and the cell suspension was passed through a 100 μm cell strainer (10282631, Corning Falcon) to obtain a single cell suspension. CD8 + T cells were isolated by negative selection using the EasySEP™ Mouse CD8 + T Cell Isolation Kit (19853, Stemcell Technologies), according to manufacturer’s instructions. The CD8 + T cells were labeled with 3 μM CFSE for 10 min at RT using the CellTrace™ CFSE Cell Proliferation Kit (C34554, Invitrogen). For the OT-I assay, 25 × 10^3^ GM–CSF DCs were plated and treated with OVA (80 μg/ml), Matrix C ISCOMs (400 ng/ml) and/or PERK inhibitor GSK2606414 (5 or 10 μM) as indicated for 5 h at 37 °C with 5% CO_2_. DCs were washed and 50 × 10^3^ CFSE-labeled CD8 + OT-I T cells (1:2 DC:T cell ratio) were added and co-culture was incubated for 24 or 72 h. Non-adherent cells were harvested and washed with FACS buffer, stained with CD8α–V450 (1:100, Clone 53–6.7, 560,469, BD), CD90.1–BV510 (1:200, Clone OX-7, 202,535, Biolegend), CD69-PE (1:100, Clone H1.2F3, 104508, biolegend), CD62L-PerCP (1:400, Clone MEL-14, 104430, Biolegend), CD44-PE/Cy7 (1:600, Clone IM7, 103,030, biolegend) and CD25-APC (1:400, Clone PC61.5, 17-0251-82, eBioscience) in FACS buffer for 20 min on ice, washed and measured on the flow cytometer Cytoflex LX (Beckman Coulter). All T cell analyses were performed after CD8 + and CD90.1 + gating. After 24 h of co-culture CD69 and CD25 were analyzed and after 72 h CD44, CD62L and CFSE proliferation were analyzed. Cell-free supernatant after 3 days of co-culture was temporarily stored at − 80 °C and IFN-γ levels were measured using the IFN gamma Mouse Uncoated ELISA Kit (88-7314, Invitrogen) according to manufacturer’s instructions.

### Lipid body stainings

GM–CSF-cultured BMDCs were sorted in CD11c + MHCII^lo^CD11b^hi^ and CD11c + MHCII^hi^CD11b^int^ BMDCs. MHCII^lo^CD11b^hi^ BMDCs were plated with 75 × 10^3^ cells per well on Fibronectin-coated (coated with 20 μg/ml in PBS for 1 h at RT, 11080938001, Roche) Chamber Slides (734-2050, Nunc™ Lab-Tek™ II), were untreated (control) or treated with Matrix C ISCOMs (400 ng/ml), oleic acid (50 μM), and/or the PERK inhibitor GSK2606414 (10 μM) and incubated for 5 h in the chamber slides. MHCII^hi^CD11b^int^ BMDCs were plated with 75 × 10^3^ cells per well on a 48 wells plate (CLS3548-100EA, corning costar), were untreated (control) or treated with Matrix C ISCOMs (400 ng/ml), oleic acid (50 μM), and/or the PERK inhibitor GSK2606414 (10 μM) and incubated for 5 h. MHCII^hi^CD11b^int^ BMDCs were harvested, washed in PBS and plated on Poly-l-lysine hybrobromide coated (coated with 100 μg/ml in H_2_O for 5 min at RT, P1524, Sigma-Aldrich) chamber slides and rested for 45 min on ice to allow cell attachment. Bulk GM–CSF-cultured BMDCs and sorted BMDCs without stimulation (“0 h” samples) were directly plated on Poly-l-lysine coated chamber slides and rested 45 min on ice. Chamber slides were washed with PBS, and cells were fixed with 4% PFA for 15 min at RT, and washed in PBS. LBs were stained with Bodipy™ 493/503 (7 μg/ml, D3922, Invitrogen) in PBS for 10 min at RT. Cells were washed and stained with DAPI (3 μg/ml, sc-3598, Santa Cruz Biotechnology) in PBS for 10 min at RT. DAPI was removed and chamber slides were mounted with ProLong™ diamond antifade mountant (P36961, Invitrogen), dried overnight and temporarily stored at 4 °C. Images were acquired on the Zeiss LSM900 confocal laser scanning microscope with the plan-apochromat ×63/oil DIC M27 objective. Multiple pictures were taken to analyze at least 50 cells per condition. The amount of LBs and size of LBs was determined with a script using the FIJI software, developed by Paul Rijken (Radiotherapy & OncoImmunology Laboratory, Department of Radiation Oncology, Radboud University Medical Center, Nijmegen, The Netherlands).

### Statistical analysis

In all figures, results are expressed as mean values from biological replicates with standard error of the mean (SEM). Statistical analysis was performed with GraphPad Prism Version 8.0.1. For the B3Z assays, two-way ANOVA and Dunnett’s multiple comparisons test were performed. For CCK8 assays statistics was performed on raw data, using repeated measurements one-way ANOVA and Tukey’s multiple comparisons test. RT–qPCR data was analyzed with a two-tailed paired *T* test (when comparing 2 conditions) or with mixed-effects analysis and Tukey’s multiple comparisons test (when comparing 4 conditions). For OT-I assays repeated measurements one-way ANOVA and Tukey’s multiple comparisons test were performed. The average amount of LBs was averaged per mouse (> 50 cells per sample) and then repeated measurements one-way ANOVA and Tukey’s multiple comparisons were used. *P* values ≤ 0.05 were considered significant. Significance is shown as: not significant *p* > 0.05, *p ≤ 0.05, **p ≤ 0.01, ***p ≤ 0.001, ****p ≤ 0.0001.

## Results

### RNA sequencing shows that the PERK pathway is upregulated in SBA-stimulated DCs

SBAs lead to enhanced cross-presentation in mouse BMDCs, but the key pathways and genes leading to this enhanced cross-presentation are poorly understood. The GM–CSF-cultured BMDCs consist of different subsets of which the MHCII^lo^CD11b^hi^ BMDCs respond to SBAs, while MHCII^hi^CD11b^int^ BMDCs do not [21] and thereby provide a model system to dissect the mode of action of SBAs. To reveal the genes and signaling pathways by which ISCOMs induce DC cross-presentation, RNA sequencing and gene expression analysis were performed for bulk GM–CSF-cultured BMDCs and sorted MHCII^lo^CD11b^hi^ and MHCII^hi^CD11b^int^ BMDCs (Supplementary Fig. 1). Bulk GM–CSF-cultured BMDCs treated with LPS or oleic acid were analyzed to identify genes that are differentially expressed upon ISCOM stimulation, but not upon TLR4 stimulation by LPS or LB induction by oleic acid. Oleic acid induces LBs, but does not induce cross-presentation. Moreover, bulk GM–CSF-cultured BMDCs were also compared to ISCOM-treated Flt3-L-cultured Clec9A+CD103+ BMDCs, since ISCOMs do not enhance cross-presentation in these DCs.

Upon ISCOM treatment > 2000 genes get differentially expressed in the bulk BMDCs (Fig. 1a, left) and 493 in the MHCII^lo^CD11b^hi^ BMDCs (Fig. 1a, middle). On the contrary, only 115 genes get differentially expressed in the MHCII^hi^CD11b^int^ BMDCs (Fig. 1a, right). The higher amount of differentially expressed genes (DEGs) in the MHCII^lo^CD11b^hi^ BMDCs is in line with what we have shown in our previous work, that indeed only the MHCII^lo^CD11b^hi^ BMDCs respond to ISCOM stimulation [21].

Gene Ontology analysis of the DEGs upon ISCOM stimulation in the MHCII^lo^CD11b^hi^ BMDCs shows multiple biological processes are enriched (Fig. 1b). Intriguingly, within the 15 most enriched processes, two of them are related to stress responses, including ER stress. ER stress is one of the processes that lead to activation of the UPR. This prompted us to further look into the expression of UPR related genes. Indeed, key genes of the UPR pathway PERK were significantly upregulated upon ISCOM stimulation in the bulk BMDCs and in the MHCII^lo^CD11b^hi^ BMDCs (Fig. 1a).

Next, we compared gene expression between different conditions in bulk BMDCs to find genes that are specifically ISCOM regulated using a combinatorial approach. We compared genes differentially expressed by ISCOMs, but not by TLR4 ligand LPS or LB inducer oleic acid and not differentially expressed by ISCOMs in Flt3-L-cultured BMDCs (Fig. 1c). Strikingly, the genes specifically up- or downregulated by ISCOMs (Atf3, Ddit3 and Asns) are genes linked to the UPR pathway PERK.

Since the UPR consists of three sensors and subsequent signaling pathways, i.e., PERK, IRE1α and ATF6, the gene expression for key genes of these pathways was analysed. Remarkably, the PERK pathway regulated genes Atf3, Atf4, Ddit3, Trib3, Asns and Gdf15 were all upregulated upon ISCOM stimulation in bulk BMDCs (Fig. 1d). In sorted BMDCs, this upregulation was evident in the MHCII^lo^CD11b^hi^ BMDCs but much less so in the nonresponsive MHCII^hi^CD11b^int^ subset. Genes regulated by the IRE1α (Xbp1, Erdj4) and ATF6 (BiP, Grp94) pathways of the UPR were downregulated upon ISCOM stimulation (Fig. 1d), suggesting a specific induction of the PERK pathway, rather than a general UPR induction. All in all, the RNA sequencing and gene expression analysis indicate that ISCOMs lead to an induction of the PERK pathway, specifically in the responsive MHCII^lo^CD11b^hi^ BMDCs.

### PERK inhibition blocks SBA-induced cross-presentation in DCs

**Fig. 2:**
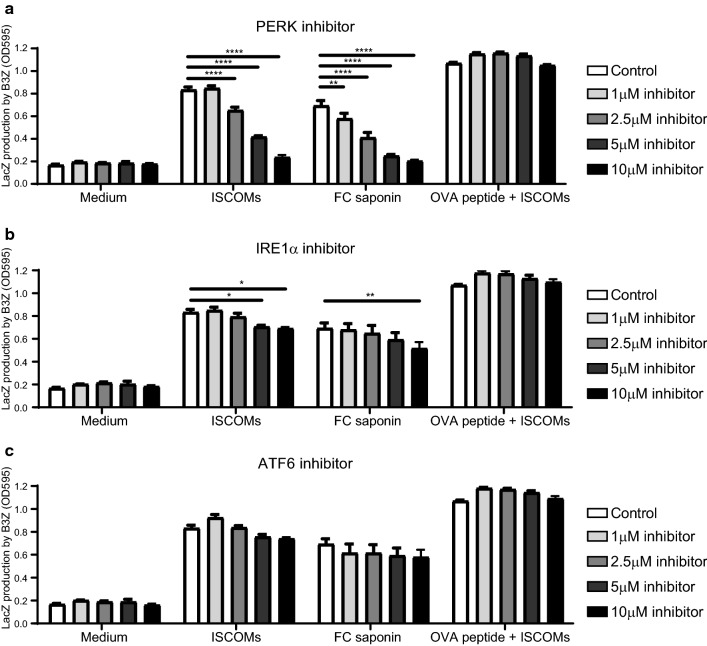
PERK blockade leads to an inhibition of SBA-induced cross-presentation in DCs. GM*–*CSF-cultured BMDCs stimulated with OVA protein are first untreated or treated with ISCOMs or FC saponin in combination with the PERK inhibitor GSK2606414 (**a**), the IRE1α inhibitor 4μ8C (**b**) or the ATF6 inhibitor PF-429242 (**c**) for 5 h and then co-cultured with B3Z T cells for 18 h. As a positive control for viability and cell surface MHC-I levels, BMDCs were pulsed with OVA peptide for 30 min before co-culture with B3Z T cells. Assays were performed with 3 biological replicates. Significance is shown as: not significant *p* > 0.05, *p ≤ 0.05, **p ≤ 0.01, ***p ≤ 0.001, ****p ≤ 0.0001

Next, we determined the role of the ER stress pathways in SBA-induced DC cross-presentation. Hence, we used the B3Z reporter T cell line, which specifically detects Ovalbumin (OVA) peptide/MHC-I complexes in a co-stimulation independent manner, to asses the level of OVA cross-presentation by GM–CSF-cultured BMDCs in the presence or absence of ISCOMs and specific UPR pathway inhibitors [21]. As expected, both FC saponin and its corresponding ISCOMs indeed induce strong DC cross-presentation of OVA protein to B3Z T cells (Fig. 2a, b, c). Subsequently, increasing amounts of the inhibitors of the PERK pathway (GSK2606414 [38]), the IRE1α pathway (4μ8C [39]) or the ATF6 pathway (PF-429242; inhibits SP1 to prevent ATF6 activation [40]) were titrated in the assay. Interestingly, blockade of the PERK pathway resulted in the inhibition of SBA-induced cross-presentation in a dose dependent manner, both for ISCOMs and FC saponin (Fig. 2a). At the concentration of 10 μM the PERK inhibitor even completely inhibited SBA-induced cross-presentation to background levels. In contrast, IRE1α inhibition only has a small effect on cross-presentation at the highest concentrations, while ATF6 inhibition has no effect on cross-presentation (Fig. 2bc). Treatment with the inhibitors does not lead to reduced cell viability or cell surface MHC-I levels, since the presentation of OVA peptide to B3Z T cells was intact in all conditions (Fig. 2). Cell metabolic activity and viability was affected by ISCOMs but not by the PERK inhibitor (Supplementary Fig. 2). In conclusion, our data indicate that the PERK pathway is critical for SBA-induced cross-presentation in a co-stimulation independent manner.

### Inhibition of cross-presentation by PERK blockade is specific for the SBA-responsive subset

**Fig. 3:**
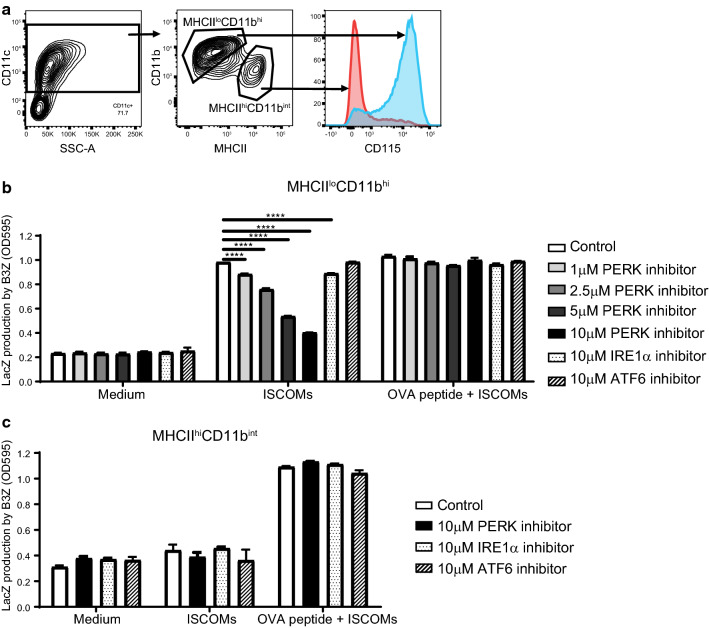
Inhibition of cross-presentation by PERK blockade is specific for the SBA-responsive subset. GM*–*CSF-cultured BMDCs were sorted into the MHCII^lo^CD11b^hi^ and MHCII^hi^CD11b^int^ subsets based on MHC-II and CD11b (**a**). Sorted cells incubated with OVA protein were treated with ISCOMs and the PERK, IRE1α or ATF6 inhibitor for 5 h and co-cultured with B3Z T cells for 18 h as a read out for cross-presentation. As a positive control for viability and cell surface MHC-I levels, BMDCs were pulsed with OVA peptide for 30 min before co-culture with B3Z T cells. B3Z assay for MHCII^lo^CD11b^hi^ BMDCs (**b**) and MHCII^hi^CD11b^int^ BMDCs (**c**). Assays were performed with 2 biological replicates and are representative for multiple experiments. Significance is shown as: not significant *p* > 0.05, *p ≤ 0.05, **p ≤ 0.01, ***p ≤ 0.001, ****p ≤ 0.0001

To investigate if the effect of the UPR inhibitors on SBA-induced cross-presentation is specific for one of the BMDC subsets, CD11c + cells were sorted into the MHCII^lo^CD11b^hi^ and MHCII^hi^CD11b^int^ subsets based on MHCII and CD11b marker expression (Fig. 3a, Supplementary Fig. 1). The subsets were treated with OVA protein, ISCOMs and the PERK, IRE1α or ATF6 inhibitors and subsequently co-cultured with the B3Z T cells. ISCOMs clearly induce DC cross-presentation and B3Z T cell activation in the MHCII^lo^CD11b^hi^ BMDCs (Fig. 3b). PERK inhibition evidently leads to inhibition of SBA-induced cross-presentation in the MHCII^lo^CD11b^hi^ BMDCs in a dose-dependent manner and complete inhibition back to background levels is seen with the 10 μM concentration (Fig. 3b). Unlike PERK blockade, IRE1α blockade only leads to a small inhibition of SBA-induced cross-presentation (Fig. 3b). ATF6 blockade does not lead to inhibition of SBA-induced cross-presentation at all (Fig. 3b). SBAs do not induce cross-presentation in the MHCII^hi^CD11b^int^ BMDCs and the UPR inhibitors do not have an effect on B3Z T cell activation (Fig. 3c). Both subsets are clearly able to passively load OVA peptide and the UPR inhibitors do not affect the MHCI levels or cell viability and thereby B3Z T cell activation levels (Fig. 3b, c). Altogether, SBAs induce cross-presentation in MHCII^lo^CD11b^hi^ but not in MHCII^hi^CD11b^int^ BMDCs. PERK inhibition completely inhibits SBA-induced cross-presentation in MHCII^lo^CD11b^hi^ BMDCs, confirming the importance of the PERK pathway for SBA-induced cross-presentation.

### DCs have a fully active UPR

**Fig. 4:**
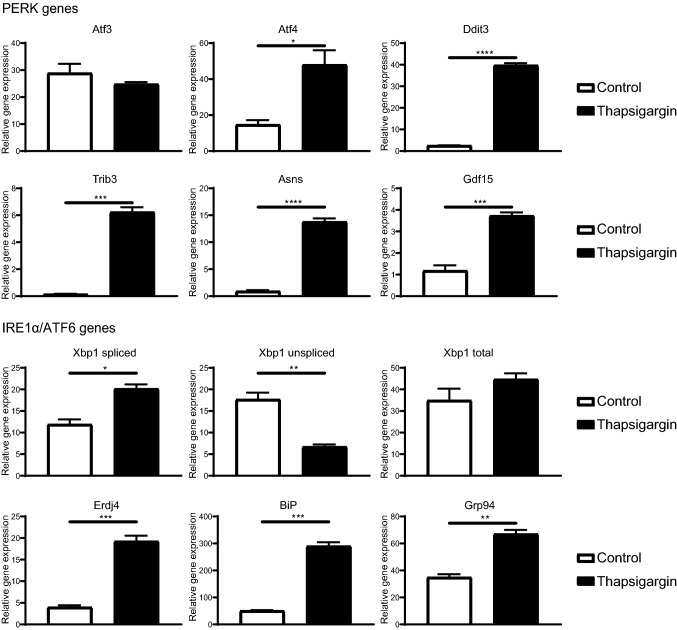
All ER stress genes are upregulated upon ER stress inducer Thapsigargin treatment. Bulk GM*–*CSF-cultured BMDCs were untreated or treated with Thapsigargin (50 nM) for 5 h. RT-qPCR was performed for mRNA expression of genes of the PERK (Atf3, Atf4, Ddit3, Trib3, Asns, Gdf15), IRE1α (Xbp1 spliced, Xbp1 unspliced, Xbp1 total expression, Erdj4) and the ATF6 (BiP, Grp94) pathways. RT-qPCR was performed with 4 biological replicates and is representative for multiple experiments. Significance is shown as: not significant *p* > 0.05, *p ≤ 0.05, **p ≤ 0.01, ***p ≤ 0.001, ****p ≤ 0.0001

RNA sequencing showed that upon ISCOM stimulation, genes part of the PERK pathway (Atf3, Atf4, Ddit3, Trib3, Asns, Gdf15) are highly upregulated in bulk BMDCs and in MHCII^lo^CD11b^hi^ BMDCs, while genes downstream of the IRE1α (Xbp1, Erdj4) and ATF6 (BiP, Grp94) pathways were mostly downregulated (Fig. 1). Next to that, PERK inhibition leads to full inhibition of SBA-induced DC cross-presentation in the bulk BMDCs and in the MHCII^lo^CD11b^hi^ BMDCs, while IRE1α and ATF6 inhibition do not (Figs. 2, 3). To investigate the capacity of the GM–CSF-cultured BMDCs to induce all three pathways downstream of ER stress and the UPR (PERK, IRE1α and ATF6), bulk BMDCs and sorted MHCII^lo^CD11b^hi^ and MHCII^hi^CD11b^int^ BMDCs were stimulated with the broad ER stress inducer Thapsigargin for 5 h and mRNA levels of UPR genes were analyzed by RT-qPCR. ER stress induction by Thapsigargin leads to upregulation of all PERK regulated genes, except Atf3, in bulk BMDCs (Fig. 4, PERK genes). ER induction by Thapsigargin also leads to activation of the IRE1α pathway shown by more Xbp1 splicing and higher Erdj4 expression, and of the ATF6 pathway indicated by higher BiP and Grp94 expression in bulk BMDCs (Fig. 4, IRE1α/ATF6 genes). Moreover, these results were reflected in both sorted subsets (Supplementary Fig. 3). This demonstrates that bulk BMDCs and sorted MHCII^lo^CD11b^hi^ and MHCII^hi^CD11b^int^ BMDCs are capable of inducing genes downstream of all UPR pathways: PERK, IRE1α and ATF6.

### SBAs induce genes downstream of PERK and PERK blockade inhibits SBA-induced Atf3 upregulation

RNA sequencing analysis showed that ISCOMs induce genes of the PERK pathway, but not of the IRE1α or ATF6 pathways. To validate these findings, sorted MHCII^lo^CD11b^hi^ and MHCII^hi^CD11b^int^ BMDCs were stimulated with ISCOMs for 5 h and mRNA levels of UPR genes were analyzed by RT-qPCR.

**Fig. 5:**
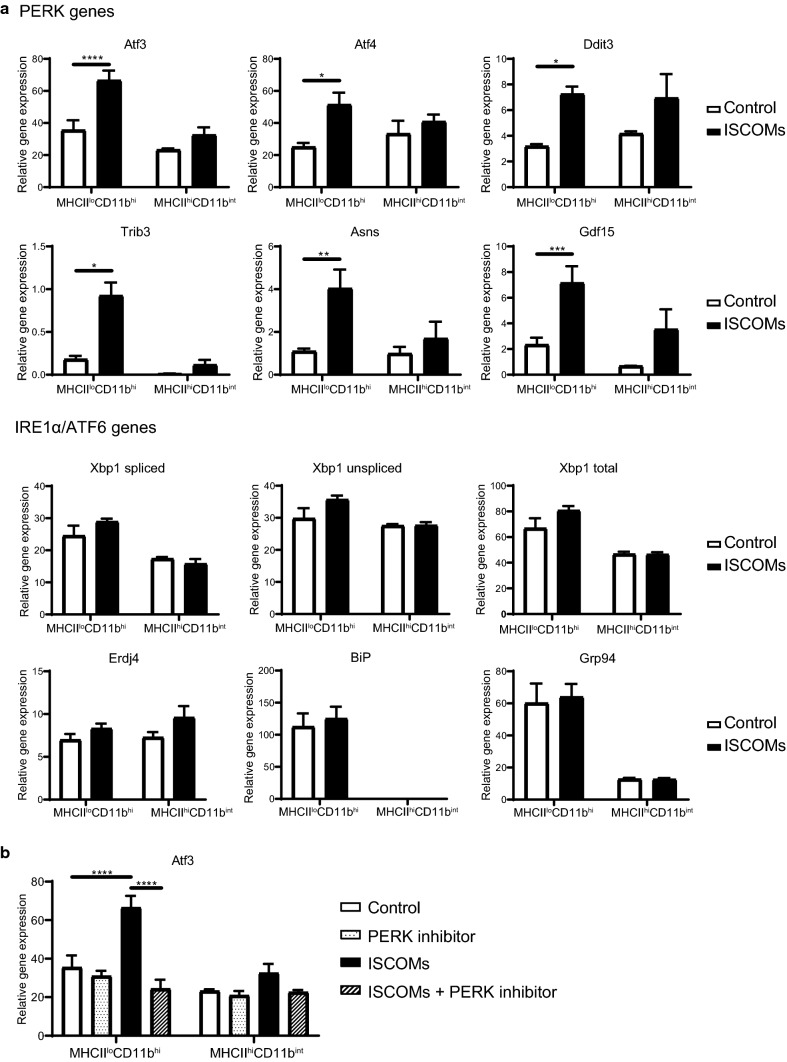
SBAs induce the PERK pathway and PERK blockade inhibits SBA-induced Atf3 upregulation. RT-qPCR for mRNA expression of genes of the PERK (Atf3, Atf4, Ddit3, Trib3, Asns, Gdf15), IRE1α (Xbp1 splicing, Xbp1 total expression, Erdj4) and the ATF6 (BiP, Grp94) pathways in the sorted MHCII^lo^CD11b^hi^ and MHCII^hi^CD11b^int^ BMDCs upon ISCOM treatment for 5 h (**a**). RT-qPCR for mRNA expression of Atf3 after treatment with ISCOMs and/or 10 μM PERK inhibitor for 5 h in the sorted MHCII^lo^CD11b^hi^ and MHCII^hi^CD11b^int^ BMDCs (**b**). RT-qPCR was performed with 2 or 3 biological replicates and is representative for multiple experiments. Significance is shown as: not significant *p* > 0.05, *p ≤ 0.05, **p ≤ 0.01, ***p ≤ 0.001, ****p ≤ 0.0001

ISCOMs lead to significant upregulation of genes of the PERK pathway, namely, Atf3, Atf4, Ddit3, Trib3, Asns and Gdf15, only in the MHCII^lo^CD11b^hi^ BMDCs, but not in the MHCII^hi^CD11b^int^ BMDCs (Fig. 5a, PERK genes). ISCOMs did not affect gene expression of the IRE1α nor ATF6 pathways, in neither of the BMDC subsets (Fig. 5a, IRE1α/ATF6 genes). While Thapsigargin induces all UPR pathways (Fig. 4), SBAs specifically induce the PERK pathway, but not the IRE1α nor ATF6 pathways. Interestingly, ISCOMs do induce gene expression of transcription factor Atf3, while ER stress induction by Thapsigargin did not induce Atf3 (Figs. 4, 5a). Since PERK inhibition leads to inhibition of SBA-induced cross-presentation, we investigated if PERK inhibition also led to inhibition of SBA-induced Atf3 mRNA expression. Sorted MHCII^lo^CD11b^hi^ and MHCII^hi^CD11b^int^ BMDCs were stimulated with ISCOMs with or without the PERK inhibitor for 5 h and mRNA levels of Atf3 were analyzed by RT-qPCR. Strikingly, the PERK inhibitor completely inhibits the ISCOM-induced expression of Atf3 in the MHCII^lo^CD11b^hi^ BMDCs (Fig. 5b). The specific induction of Atf3 by SBAs and the inhibition of SBA-induced Atf3 expression by the PERK inhibitor, suggest that Atf3 could be of importance for SBA-induced DC cross-presentation. Concluding, SBAs specifically induce genes downstream of the PERK pathway, including Atf3, in the MHCII^lo^CD11b^hi^ BMDCs but not in MHCII^hi^CD11b^int^ BMDCs. Moreover, PERK blockade leads to inhibition of SBA-induced Atf3 upregulation.

### PERK inhibition blocks SBA-induced OT-I T cell cross-priming by DCs

**Fig. 6:**
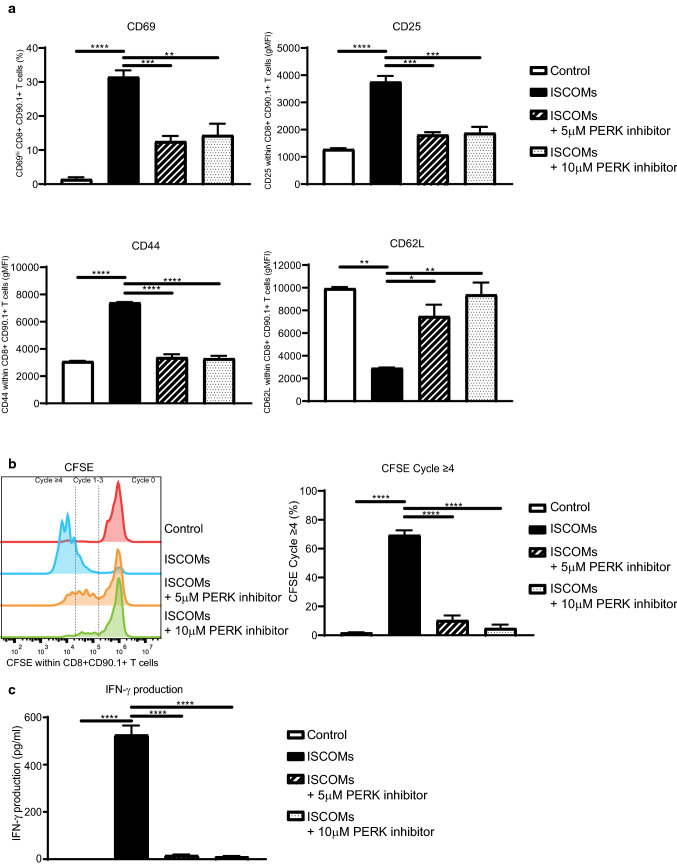
PERK inhibition blocks SBA-induced OT-I T cell activation by DCs. OT-I T cell activation assay. GM*–*CSF-cultured BMDCs were treated with OVA protein, ISCOMs and/or the PERK inhibitor, washed, and co-cultured for 24 h or 72 h with CFSE-labeled CD8 + CD90.1 + T cells isolated from OT-I transgenic mice. Marker expression within CD8 + CD90.1 + T cells with CD69 and CD25 expression after 24 h and CD44 and CD62L after 72 h of co-culture (**a**). CFSE staining as read out for proliferation within CD8 + CD90.1 + T cells (left) and the percentage of T cells which proliferated four or more times (right) after 72 h of co-culture (**b**). IFN-γ production measured in the supernatant after 72 h of co-culture (**c**). Assays were performed with 3 biological replicates. Significance is shown as: not significant *p* > 0.05, *p ≤ 0.05, **p ≤ 0.01, ***p ≤ 0.001, ****p ≤ 0.0001

Since PERK blocks SBA-induced OVA cross-presentation of DCs to B3Z T cells, we investigated the effect of SBAs in combination with PERK inhibition on T cell activation in another model system using OT-I T cells. GM–CSF-cultured BMDCs were stimulated with OVA protein and ISCOMs in combination with the PERK inhibitor for 5 h, washed and then co-cultured with CFSE-labeled CD8 + CD90.1 + T cells isolated from the spleen of OT-I transgenic mice for either 24 h or 72 h. OT-I T cells get activated upon DC cross-priming, which is, unlike the B3Z model, dependent on both DC cross-presentation leading to OVA peptide/MHC-I complexes and DC maturation (co-stimulatory molecules and cytokines). T cell activation was assessed by measuring levels of activation markers (CD69, CD25, CD44 and CD62L) and proliferation by loss of CFSE labeling using flow cytometry and IFN-γ production by ELISA. ISCOMs induce a strong activation of T cells shown by an early increase in CD69 and CD25 expression after 24 h and a later increase in CD44 expression, but a decrease in CD62L expression after 72 h of co-culture (Fig. 6a). Strikingly, the PERK inhibitor completely prevents SBA-induced CD69, CD25 and CD44 upregulation and CD62L downregulation (Fig. 6a). Moreover, ISCOM treatment lead to a high amount of proliferating T cells, whereby most T cells have proliferated four or more times after 72 h of co-culture, shown by a loss of CFSE labeling (Fig. 6b). Convincingly, PERK inhibition leads to a strong dose-dependent reduction of SBA-induced T cell proliferation (Fig. 6b). In line with this, ISCOMs lead to a strong induction of IFN-γ production by these T cells after 72 h which is completely inhibited by PERK inhibition (Fig. 6c). As shown here, PERK inhibition completely prevents SBA-induced T cell cross-priming by DCs, which underlines the crucial role of the PERK pathway in SBA-induced DC cross-presentation and cross-priming.

### PERK inhibition does not prevent SBA-induced LBs

**Fig. 7:**
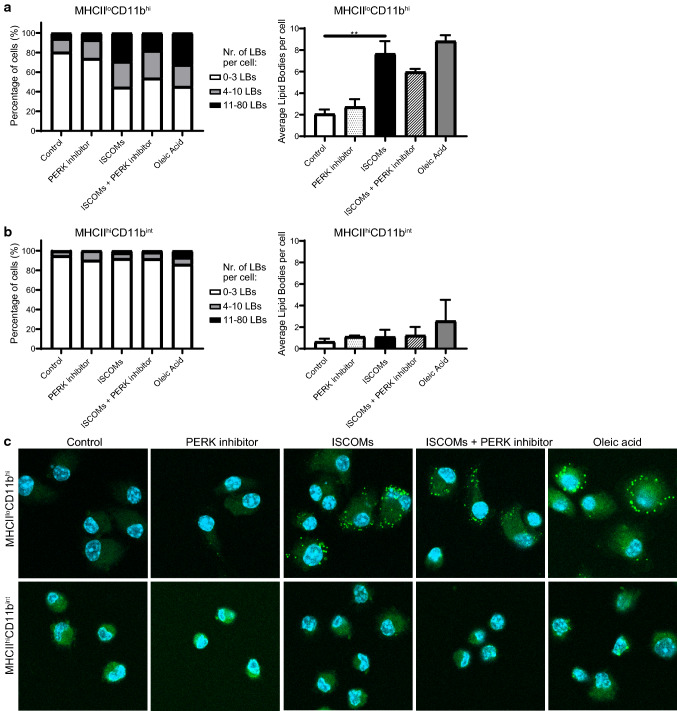
PERK inhibitor partly prevents ISCOM-induced LBs**.** Sorted MHCII^lo^CD11b^hi^ and MHCII^hi^CD11b^int^ GM*–*CSF-cultured BMDCs were untreated or treated with ISCOMs and/or the PERK inhibitor or with oleic acid for 5 h. Percentage of cells with 0*–*3, 4*–*10 or 11*–*80 LBs per cell (left) and the average amount of LBs per cell (right) for the responsive MHCII^lo^CD11b^hi^ BMDCs (**a**) and the nonresponsive MHCII^hi^CD11b^int^ BMDCs (**b**) and representative confocal images (**c**). Confocal images: nuclear DAPI in blue and BODIPY 493/503 LBs in green. LBs stainings were performed with 2 biological replicates and are representative for multiple experiments. Every condition contains > 50 cells per replicate. Significance is shown as: not significant *p* > 0.05, *p ≤ 0.05, **p ≤ 0.01, ***p ≤ 0.001, ****p ≤ 0.0001

Our previous research has shown that SBAs induce LBs, and that LBs are crucial for SBA-induced cross-presentation [21]. Moreover, SBA-induced LBs are specific for the responsive MHCII^lo^CD11b^hi^ BMDCs [21]. To investigate role of PERK pathway in LB formation, sorted MHCII^lo^CD11b^hi^ and MHCII^hi^CD11b^int^ GM–CSF-cultured BMDCs were untreated or treated with ISCOMs and/or the PERK inhibitor or with LB inducer oleic acid for 5 h. LBs were stained by Bodipy 493/503 and visualized by confocal microscopy. BMDCs had low amounts of LBs at the start of the experiment, showing that cell harvesting and sorting did not affect the amounts of LBs in this assay (Supplementary Fig. 4). Indeed, ISCOMs clearly induce many LBs in a high amount of cells in the MHCII^lo^CD11b^hi^ BMDCs, with more than 25% of cells having 4–10 LBs and more than 25% of cells having even 11–80 LBs (Fig. 7a). In the MHCII^lo^CD11b^hi^ BMDCs the average amount of LBs is significantly increased upon ISCOM treatment (Fig. 7b), which can be seen in the representative pictures (Fig. 7c). In addition, the LB inducer oleic acid leads to high amounts of LBs in MHCII^lo^CD11b^hi^ BMDCs (Fig. 7ac). In the MHCII^lo^CD11b^hi^ BMDCs, treatment of ISCOMs combined with the PERK inhibitor does not significantly affect the average amount of LBs (Fig. 7a, right); however, the percentage of cells with 11–80 LBs per cell is somewhat lower compared to ISCOM-stimulated cells (Fig. 7a, left). In the MHCII^hi^CD11b^int^ BMDCs, ISCOM and oleic acid treatment leads to LB induction in a small amount of cells and PERK inhibition does not affect this (Fig. 7bc). In conclusion, PERK activation and LB formation are both crucial for SBA-induced cross-presentation.

## Discussion

SBAs have been shown to be outstanding adjuvants in pre-clinical and clinical studies for both cancer and viral infections, but the underlying mechanisms are still poorly understood [4, 6, 41–46]. Especially the mechanisms underlying the effect of SBAs on DC cross-presentation in the MHCII^lo^CD11b^hi^ BMDCs remain incompletely understood. Here we showed that SBAs induce the PERK pathway of the UPR, specifically in the MHCII^lo^CD11b^hi^ BMDCs. Inhibition of the PERK pathway completely inhibits SBA-induced cross-presentation and CD8 + T cell activation in this DC subset.

Our B3Z and OT-I assays show that PERK is crucial for SBA-induced DC cross-presentation in the MHCII^lo^CD11b^hi^ BMDCs. However, the precise manner by which PERK contributes to SBA-induced cross-presentation remains to be elucidated. IRE1α and PERK of the UPR are constitutively expressed and activated in CD8α + DCs but not in CD11b + DCs [47]. Loss of Xbp1 (downstream of IRE1α) leads to impaired cross-presentation of cell-bound antigens by CD8α + DCs, while inducing Xbp1 leads to improved cross-presentation [47, 48]. In the CD8α + DCs, loss of Xbp1 also leads to downregulation of PERK. Interestingly, in the CD11b + DCs no effects were observed upon loss of Xbp1. Several studies have shown that activation of PERK and expression of Atf4 and Ddit3 can contribute to DC activation [49–51]. This is all in line with our findings that in the MHCII^lo^CD11b^hi^ BMDCs, which have a low constitutive UPR signaling, UPR induction can lead to improved cross-presentation.

Several groups have shown that ERAD plays a role in DC cross-presentation by enabling antigen dislocation and that blocking ERAD, or specific ERAD members, such as Sec61 or AAA + ATPase p97, lead to a repression of cross-presentation [14–19]. Interestingly, Shoulders et al. show ERAD induction upon IRE1α and ATF6 activation, while PERK activation was not studied [52]. SBAs specifically induce the PERK pathway and a role of ERAD cannot be ruled out based on our data.

The transcription factor Atf3 is induced in DCs upon SBA treatment, while PERK blockade inhibited DC cross-presentation and Atf3 induction. Atf3 is a stress-induced transcription factor which can form homodimers, but also heterodimers with other transcription factors, such as ATF2, c-Jun, JunB and JunD, and plays a vital role in modulating metabolism and immunity [53]. In myeloid cells, Atf3 becomes activated upon TLR stimulation, IFN stimulation or bacterial infection and either represses or induces cytokine production depending on the study design. Atf3 is also shown to repress pro-apoptotic genes Bax and Bak [54–57]. Thus, Atf3 could possibly prevent apoptosis induced by PERK activation upon SBA treatment or regulate cytokine responses.

Next to the role of storing lipids, LBs also can have other functions and their content is crucial for the DC’s behavior and cross-presenting capacity [23]. In several studies LBs have been shown to be necessary for DC cross-presentation [21, 25, 26, 32], while in others LB presence blocks DC cross-presentation [24, 27–31], suggesting that the LB content is more important than just the presence of LBs by itself. Next to the lipid content of LBs, also the proteins on the LB membrane could play an important role. Both ADRP and IGTP are associated with LBs and are crucial for the LB induction and cross-presenting ability of DCs upon SBA treatment [21], but also upon IFN-γ treatment [25]. We showed that LB formation and PERK activation are both crucial for SBA-induced cross-presentation. No major impact on LB induction by PERK inhibition was observed, although some reduction in cells with a high amount of LBs was found. It is clear that components of the UPR play a role in the regulation of lipid metabolism [58] and that ER stress can lead to LB induction [31, 59]. How SBA-induced LBs differ from other LBs and if inhibition of PERK affects the LB content or LB membrane protein expression still needs to be investigated further.

Investigating the mechanisms of SBA-induced DC cross-presentation will contribute to the knowledge about cross-presentation specifically in the MHCII^lo^CD11b^hi^ DC subset, which is not widely studied. As antigen cross-presentation is key for the potency of vaccine adjuvants, knowledge of SBAs’ mechanisms will contribute to vaccine development. SBAs are derived from the South American soapbark tree, *Quillaja Saponoria*. Ultimately, compound(s) mimicking SBAs’ mode of action will allow for large scale production and development of an off-the-shelf product.

Concluding, our data show that PERK activation is crucial for SBA-induced DC cross-presentation. Understanding the mechanisms of SBA adjuvant activity will stimulate the development of new and improved vaccines enhancing DC cross-presentation and CD8 + T cell immunity.

## Supplementary Information

Below is the link to the electronic supplementary material.

Supplementary Fig. 1 Gating strategy for cell sorting. Gating strategy for cell sorting of GM–CSF-cultured CD11c+ BMDCs sorted into the MHCII^lo^CD11b^hi^ and MHCII^hi^CD11b^int^ BMDC subsets based on CD11c, MHC-II and CD11b expression. Representative of multiple experiments

Supplementary Fig. 2 The PERK inhibitor does not affect cell viability. CCK8 assay as a read out for cell viability and metabolic activity. GM–CSF-cultured BMDCs are untreated or treated with ISCOMs and/or the PERK inhibitor for 5h, washed and untreated for 18 h (timing as in B3Z assay). The relative metabolic activity was calculated as (treatment—blank)/(control—blank)x100%. Assays were performed with 3 biological replicates. Significance is shown as: not significant *p* >0.05, *p ≤0.05, **p ≤0.01, ***p ≤0.001, ****p ≤0.0001

Supplementary Fig. 3 mRNA expression profiles upon Thapsigargin treatment. Sorted MHCII^lo^CD11b^hi^ and MHCII^hi^CD11b^int^ GM–CSF–cultured BMDCs were untreated or were treated with Thapsigargin (50nM) for 5h. RT-qPCR was performed for mRNA expression of genes downstream of the PERK (Atf3, Atf4, Ddit3, Trib3, Asns, Gdf15), IRE1α (Xbp1 splicing, Xbp1 total expression, Erdj4) and the ATF6 (BiP, Grp94). RT-qPCR was performed with 2 biological replicates of MHCII^lo^CD11b^hi^ and 1 biological replicate of MHCII^hi^CD11b^int^ BMDCs and is representative for multiple experiments

Supplementary Fig. 4 LBs before stimulation. Percentage of cells with 0–3, 4–10 or 11–80 LBs per cell and their representative confocal images for bulk BMDCs and for MHCII^lo^CD11b^hi^ and MHCII^hi^CD11b^int^ BMDCs before stimulation (0h). Confocal images: nuclear DAPI in blue and BODIPY 493/503 LBs in green. LB stainings were performed with 2 biological replicates and are representative for multiple experiments. Every condition contains > 50 cells per replicate

## Data Availability

The RNA sequencing data sets generated and analysed during the current study are not publicy available, but are available from the corresponding author on reasonable request.

## Abbreviations

ATF6: Activating transcription factor 6α
BMDC: Bone-marrow-derived dendritic cell
DC: Dendritic cell
DEGs: Differentially expressed genes
ERAD: ER-associated degradation
FC: Fraction C
IRE1α: Inositol-requiring enzyme 1α
ISCOMs: Immune stimulatory complexes
LB: Lipid body
OVA: Ovalbumin
PERK: PKR-like endoplasmic reticulum kinase
SBA: Saponin-based adjuvant
UPR: Unfolded protein response
